# Potent and Targeted Sindbis Virus Platform for Immunotherapy of Ovarian Cancer

**DOI:** 10.3390/cells12010077

**Published:** 2022-12-24

**Authors:** Silvana Opp, Alicia Hurtado, Christine Pampeno, Ziyan Lin, Daniel Meruelo

**Affiliations:** Department of Pathology, NYU Grossman School of Medicine, New York University, New York, NY 10016, USA

**Keywords:** oncolytic virus vaccine platform, Sindbis, alpha-virus immunotherapy, cancer immunity, anti-tumor immunity, ovarian cancer immunotherapy

## Abstract

Our laboratory has been developing a Sindbis viral (SV) vector platform for treatments of ovarian and other types of cancers. In this study we show that SV.IL-12 combined with an agonistic OX40 antibody can eliminate ovarian cancer in a Mouse Ovarian Surface Epithelial Cell Line (MOSEC) model and further prevent tumors in mice rechallenged with tumor cells after approximately 5 months. Treatment efficacy is shown to be dependent upon T-cells that are transcriptionally and metabolically reprogramed. An influx of immune cells to the tumor microenvironment occurs. Combination of sequences encoding both IL-12 and anti-OX40 into a single SV vector, SV.IgGOX40.IL-12, facilitates the local delivery of immunoregulatory agents to tumors enhancing the anti-tumor response. We promote SV.IgGOX40.IL-12 as a safe and effective therapy for multiple types of cancer.

## 1. Introduction

Ovarian cancer (OC) is the most lethal of all gynecological malignancies. It is characterized by a unique tumor microenvironment (TME) that facilitates metastasis, impairs immune surveillance, and mediates therapy resistance [1]. In 2022, the numbers of estimated cases and deaths for of OC are 19,880 and 12,810, respectively [2]. While the overall 5-year survival rate has remained ~50% for the past decade, only 30.8% of women with distant metastatic cancer will survive [2]. New treatments are urgently needed.

Our laboratory has been developing a Sindbis viral vector platform for treatments of ovarian and other types of cancers [3,4,5,6,7]. Sindbis virus (SV), an enveloped, single-stranded, positive sense RNA virus, is a member of the alphavirus genus [8]. Several alphavirus vectors have been designed to express genes of interest in mammalian cells for vaccine, cancer, and gene therapies [9].

Attributes of alphaviruses that render them advantageous for vector systems include: (1) a broad host range for entry into mammalian cells [10]; (2) formation of a replication complex in the cytoplasm of infected cells that produces approximately 10^6^ copies of viral RNA, which coupled with a strong subgenomic promoter, enables very high expression levels of recombinant protein [11,12]; (3) RNA replication without DNA intermediates avoids risk of chromosomal integration or insertional mutagenesis; (4) transmitted via insect bites, alphaviruses are blood-borne and, hence, recombinant vectors can be systemically delivered [8]; (5) alphaviruses are known to target lymph nodes [13]; and (6) the double-stranded RNA replication intermediate acts as a danger signal to stimulate innate immune responses [14].

Sindbis virus (SV) has the safest profile among alphaviruses. Mostly asymptomatic infections occur that can lead to mild fever, rash, and arthralgia that promptly resolves [15,16,17]; more rarely, in some DRB1*01-positive individuals, arthritic symptomology can persist longer [18]. To avoid even transient adverse effects, our vectors have been attenuated by splitting the SV genome and removing the packaging signal from the genomic strand that encodes the structural genes [11,12,19] resulting in propagation-defective vectors.

Using SV vectors that express bioluminescent or enzymatic reporter genes, we have shown, by light imaging and histological detection, respectively, that SV vectors can specifically target and suppress the growth of tumor and metastatic cells in both murine xenotropic and syngeneic ovarian cancer models [3,4]. The tumor specificity of SV vectors correlates with the binding of SV to the cell surface 67-kDa high-affinity laminin receptor [20,21], which is overexpressed on several types of human cancer cells [22,23,24,25,26,27,28], including those of ovarian origin [28]. We have shown that C.B-17-SCID mice, injected with the human cell line, ES-2 clear-cell ovarian carcinoma [29], in which laminin receptor expression was knocked down with RNAi, results in tumors that are significantly less infected by the SV vector compared with the parental ES-2 cells.

SV vector cancer therapy can involve several processes. Cytotoxic effects of SV infection result from inhibition of cellular protein translation, activation of a stress response, and ultimately an apoptotic cascade [29]. Early in our investigations we observed that SV vector expression of IL-12 [4] and IL-15 [5] cytokines enhanced anti-tumor activity. The roles of IL-12 and NK cell activation were studied in the ES-2, xenotropic, C.B-17-SCID mouse ovarian cancer model, which lacks T and B cells. The therapeutic effects of SV vectors expressing IL-12 were found to be dependent upon the cytotoxic and regulatory functions of natural killer (NK) cells, which migrated to the peritoneum of mice with ES-2 tumors [30].

The potential of SV vectors to stimulate an adaptive immune response against tumors was studied in a syngeneic, immunocompetent BALB/c CT26 colon carcinoma tumor model [31]. The oncolytic effects of SV vector treatment were precluded as CT26 cells are not susceptible to SV infection. Within 3 h after intraperitoneal (i.p.) injection, an SV bioluminescent reporter vector produced signals within the mediastinal lymph nodes that drain the peritoneum. When SV vectors that present tumor-associated antigens (TAA) were injected into tumor-bearing mice, a large influx of activated CD8+ T cells to the peritoneum occurred within 1 week. SV vector treatment led to long-term survival correlating with the generation of antigen specific CD8+ effector and memory T cells. It was also shown that oncolysis of tumor cells produced immune activation against endogenous tumor antigens by a process known as epitope spreading [32], suggesting the possibility that SV vectors can be therapeutic without specific tumor targeting.

Immunotherapeutic effects of the SV vector system were tested by expression of immunomodulatory proteins such as cytokines [4,5,30], check-point inhibitors [6], or co-stimulatory antibodies [7] alone or in multiple combinations. The results of our extensive studies in multiple preclinical models indicated that the SV.IL-12 vector in combination with the agonistic antibody (Ab) for OX40 was the most efficacious and was devoid of any adverse effects [7].

OX40 is a TNF-family co-stimulatory receptor that is expressed on activated T cells [33] and also functions to repress regulatory T cell induction [34]. Activated OX40 promotes clonal expansion, differentiation, survival of Th1 effector T cells and induction of Th-1-type cytokines such as local interferon gamma (IFNγ) and IL-2 secretion [35,36,37,38,39,40]. OX40 controls survival of primed CD8+ T cells and confers CTL-mediated protection by regulating Th-1-type T helper cell functions [35,41]. In addition, OX40 is co-expressed with ICOS on transitional and mature follicular T helper (TFH) cells [42], a subset of CD4+ T helper cells that secrete IL-21 and facilitate the differentiation of Ab producing B cells and long-lived plasma cells from germinal center [43] B cells. OX40 stimulation is found to cooperate with ICOS-ICOSL interactions to maintain TFH responses after the initial TFH phenotype is acquired and it amplifies an ongoing Ab response [44].

IL-12 activates T cells and increases the expression of OX40 on effector CD4 T cells [45], which can explain the synergy between these two immunomodulators [7]. IL-12 also stimulates the production of IFNγ, which induces and maintains higher levels of anti-tumor macrophages [46,47]. Inhibition of tumor angiogenesis and release of matrix metalloproteinases have also been attributed to IL-12 signaling [48,49,50].

At least 25 different IL-12-expressing oncolytic vectors (OV-IL12s) have been genetically engineered for local IL-12 production and preclinically tested in various cancer models. Among OV-IL12s, oncolytic herpes simplex virus encoding IL-12 (OHSV-IL-12) is the furthest along in the clinic [51]. Expression of IL-12 and its application as a monotherapy, however, does not generally provide a desired therapeutic outcome, as demonstrated in several preclinical cancer models; it requires a synergistic or additive combination approach with other anti-cancer therapies for an improved therapeutic outcome [52,53,54,55,56,57,58].

Currently, there are no trials listed in ClinicalTrials.gov that involve both αOX40 and IL-12 for cancer therapy. One adenovirus, DNX-2440, a replication-competent adenovirus that expresses the human OX40 ligand is being tested against recurrent glioblastoma (Clinical Trials.gov identifier: NCT03714334) and colorectal and other cancers with liver metastasis (NCT04714983). A herpes simplex [59] virus 1-based OV encoding the OX40 ligand and IL-12 is being preclinically tested in combination with tumor-infiltrating lymphocyte therapy [59].

In this study we show that SV.IL-12 combined with an agonistic OX40 antibody can eliminate ovarian cancer in a mouse model and further prevent tumors in mice rechallenged with tumor cells after approximately 5 months. Treatment efficacy is shown to be dependent upon T cells that are transcriptionally and metabolically reprogrammed. An influx of immune cells to the tumor microenvironment occurs. Combination of sequences encoding both IL-12 and anti-OX40 into a single SV vector, SV.IgGOX40.IL-12 facilitates the local delivery of immunoregulatory agents to tumors enhancing the anti-tumor response.

## 2. Materials and Methods

### 2.1. Cell Lines

Baby hamster kidney (BHK-21) cells were obtained from the American Type Culture Collection. ID8 Mouse Ovarian Surface Epithelial Cell Line (MOSEC) were a generous gift of Dr. Katherine F. Roby, University of Kansas Medical Center, Kansas City [60]. MOSEC ID8 is frequently used as a syngeneic mouse model for high grade serous ovarian cancer (HGSOC). Firefly-luciferase (Fluc)-expressing (MOSEC) were generated by stable transfection of pGL4.20_Fluc plasmid and further 11 in vivo passaging produced a more stable cell line in mouse model that we termed MOSEC.Fluc.p11 cells. 

BHK-21 cells were maintained in α-modified minimum essential medium (α-MEM) (Corning Cellgro, Manassas, VA, USA) with 5% fetal calf serum (FCS, Gibco) and 100 mg/mL penicillin/streptomycin (Corning Cellgro, Manassas, VA, USA). MOSEC.Fluc.p11 cells were maintained in Dulbecco’s modified Eagle’s medium (DMEM) containing 4.5 g/L glucose (Corning Cellgro, Manassas, VA, USA) supplemented with 4% FCS, 100 mg/mL penicillin/streptomycin and 1× Insulin-transferrin-sodium selenite (ITS supplement, Gibco, Grand Island, NY, USA). All cell lines were cultured at 37 °C and 5% CO_2_.

### 2.2. SV Production

All SV vectors used in these studies are propagation defective and were produced as previously described [7,19,61]. SV empty vector is the same plasmid without an additional gene of interest (e.g., IL-12 or IgGOX40). Briefly, plasmids carrying the replicon (e.g., SV.IL-12, SV.IgGOX40 and SV.IgGOX40.IL-12) or Sindbis helper RNAs were linearized with *XhoI*, in vitro transcribed, electroporated into BHK-21 cells, harvested from media, and titrated.

Sequence of the agonistic OX40 antibody is published in patent application number WO2021/007276 A1. The IL-12 sequence is a synthetic construct of bioactive single-chain murine interleukin 12 mRNA, complete cds Genbank Sequence ID: AF411293.1.

### 2.3. Animal Experiments and Tumor Models

All experiments were performed in accordance with the Institutional Animal Care and Use Committee of New York University Health. Six- to twelve-week-old female C57BL/6 albino mice, B6(Cg)-*Tyr^c−2J^*/J, were purchased from Jackson Laboratory (stock no.: 000058).

#### 2.3.1. Tumor Injection and Treatments

MOSEC.Fluc.p11 tumor cells, 2.5 × 10^6^ in 0.5 mL opti-MEM (Gibco, Grand Island, NY, USA) per mouse, were i.p. inoculated on day 0. For treatments, mice were randomized and SV vector (10^7^ transduction units (TU)/mL), in a total of 0.5 mL was i.p. injected into the left side of the animal four times each week for a total of 4 weeks, starting on day 7 unless otherwise indicated. Tumor burden was monitored using 2D in vivo imaging system (IVIS) bioluminescence imaging. Briefly, mice were injected with 150 mg/kg D-luciferin Firefly, potassium salt (GoldBio, St. Louis, MO, USA), and luminescence was assessed 5 min later using the Caliper Life Sciences Imaging System. Images were analyzed with Living Image Software 3.0 software. Relative tumor growth was calculated individually for each animal dividing the total body bioluminescence signal of a given day by the first one registered before treatment (day X/ first day). As per indicated treatment strategy, mice were injected i.p. with indicated doses of SV vectors and/or commercial αOX40 antibody (dose-250 µg/mouse, BioXcell, Lebannon, NH, USA; BE0031). Control mice were injected with either PBS or isotype control antibody. Treatments were repeated for SV vectors i.p. four times a week for a total of 4 weeks and for αOX40 antibody three times a week i.p. for a total of 3 weeks. Mice were monitored for tumor growth and survival. For rechallenge experiments, 2.5 × 10^6^/mouse MOSEC.Fluc.p11 cells were i.p. injected.

#### 2.3.2. Depletion of CD8 and CD4 T Cells In Vivo

In vivo depletion of CD8 or CD4 T cells was achieved by i.p. injection of 100 µg InVivoMAb anti-mouse CD8 (BioXcell, Lebannon, NH, USA, BE0061) or 500 µg InVivoMAb anti-mouse CD4 (BioXcell, Lebannon, NH, USA; BE0003-1) per mouse. For the isotype control, 500 µg of IgG2b (BioXcell, Lebannon, NH, USA; BE0090) was i.p injected per mouse. αCD4, αCD8 and isotype control were administered starting on day 1 and day 4 after tumor inoculation, following an administration every 4 days for 4 weeks. Groups were treated i.p. with SV.IgGOX40.IL-12 starting on day 5 of the experiment, 4 times per week, for 4 weeks in total. Mice were monitored for tumor growth and survival.

### 2.4. RNA-Seq

#### 2.4.1. mRNA Library Prep

Total RNA was extracted from freshly isolated tumors on day 14 of treatment using the RNeasy kit (Qiagen, Germantown, MD, USA). For each group, three mice were used for biological repeats. RNA sequencing was performed by NYU Langone Genome Technology Center. RNA extractions were quantified using RNA Nano Chips (Agilent Tech., Inc., Santa Clara, CA, USA, Cat. #5067-1511) on an Agilent 2100 BioAnalyzer. RNA-Seq library preps were constructed using the Illumina TruSeq^®^ Stranded mRNA Library Prep kit (Illumina, Inc., San Diego, CA, USA, Cat #20020595) using 150ng of total RNA as input with 13 cycles of PCR amplification. Sequencing was paired-end 50 cycles using an SP100 cycle flowcell-v1.5 on an Illumina NovaSeq6000 sequencer with 2% PhiX spike-in.

#### 2.4.2. Data Analysis

(https://github.com/igordot/sns/blob/master/routes/rna-star.md (accessed on 11 December 2022)). Adapters and low-quality bases were trimmed using Trimmomatic (v0.36) [62]. Sequencing reads were mapped to the reference genome (mm10) using the STAR aligner (v2.7.3) [63]. Alignments were guided by a gene transfer format (GTF) file. The mean read insert sizes and their standard deviations were calculated using Picard tools (v.2.18.20) (http://broadinstitute.github.io/picard (accessed on 11 December 2022)). The gene sample counts matrix was generated using FeatureCounts (v1.6.3) [64,65] and normalized based on their library size factors using DEseq2(v1.30.1) [66], and differential expression analysis was performed. To compare the level of similarity among the samples and their replicates, we used principal-component analysis [67]. Gene set enrichment analysis (GSEA) [68] of differentially expressed genes (DEGs) from control versus SV vector treatments was performed. STRING Functional Protein Association Networks Tool [69] was used to investigate the modulation of anti-tumor immune responses by gene of interest SV vector expressing therapies.

### 2.5. Measurement of OCRs and ECARs of T Cells

T-cell metabolic output was measured by Seahorse technology as previously described [7,70]. Purified T cells were plated at 6 × 10^5^ cells/well in an Agilent Seahorse XF24 cell culture microplate. Oxygen consumption rates (OCRs) and extracellular acidification rates (ECARs) were measured using an Agilent Seahorse XFe24 metabolic analyzer following the procedure recommended by the manufacturer (Agilent Technologies, Santa Clara, CA, USA). For the mitochondrial stress test, (1) oligomycin (1 μM), (2) carbonyl cyanide 4-(trifluoromethoxy)phenylhydrazone (FCCP) (1.5 μM), and (3) rotenone (100 nM) and antimycin A (1 μM) were injected sequentially through ports A, B, and C.

### 2.6. Histochemistry and Multiplex Immunofluorescence (MIF)

Tumors of mice were collected, fixed in 4% paraformaldehyde (PFA) for 2 days, embedded in paraffin, sectioned, and H&E stained. For MIF staining and imaging, 5 μm paraffin sections were stained with an Akoya Biosciences Opal multiplex automation kit (Akoya Biosci., Marlborough, MA, USA; 01752) on a Leica BondRX autostainer, according to the manufacturers’ instructions. Prior to incubation with the first primary antibody, sections underwent heat retrieval with Bond Epitope Retrieval Buffer 2 (Leica ER2, AR9640, Leica biosystems, Nussloch, GmbH) and blocking. Primary antibodies were against CD4 (1:500, Cell Signaling Technology, Danvers, MA, USA; 25229), CD8 (1:2000, Cell Signaling Technology, Danvers, MA, USA; 98941S), Ki-67 (1:200, Abcam, Cambridge, United Kingdom; AB16667), and granzyme B (1:1000, Abcam, Cambridge, United Kingdom; AB4059). Each primary antibody was followed by a cocktail of horseradish-peroxidase-conjugated secondary antibodies against mouse and rabbit immunoglobulin G (IgG) (RTU, Akoya/PerkinElmer, Hopkinton, MA, USA; catalog no. ARH1001) and then tyramide-mediated signal amplification (TSA) with covalent linkage of the individual Opal fluorophore (each 1:250, Opal 520 [FP1496001KT], 540 [FP1487001KT], 570 [FP1494001KT], 620 [FP1488001KT], 650 [FP1495001KT], or 690 [FP1497001KT], Akoya/PerkinElmer [catalog nos.]) to the tissue antigen. Antibodies were subsequently stripped using either ER1 (Leica, AR9961) or ER2 (Leica, AR9640) heat retrieval buffer, and the next round of immunostaining was initiated. After completion of the sequential incubations and stripping, slides were counterstained with spectral DAPI (Akoya/PerkinElmer, Hopkinton, MA, USA; FP1490). Monoplex controls were used to confirm appropriate staining for antibodies integrated into the multiplex panels. Multispectral imaging was performed on a Vectra3 imaging system (Akoya/PerkinElmer, Hopkinton, MA, USA) at ×20. The fluorophore emission signatures were captured by a multispectral camera and then unmixed with INFORM software (Akoya/PerkinElmer, Hopkinton, MA, USA). Autofluorescence, obtained from an unstained slide, was removed from the composites and pseudo-colored images were exported as tif files.

### 2.7. Statistical Analysis

Statistical analysis was performed using GraphPad Prism 9.0 as described in the figure legends. All data are shown as mean ± SEM. Figures were prepared using GraphPad Prism 9, Adobe Photoshop, and ImageJ software. Treated groups were compared, with a one-way analysis using GraphPad Prism 8, to naive mice. Differences with a *p* value of <0.05 were considered significant: * *p* < 0.05, ** *p* < 0.005, *** *p* < 0.0001. *p* values were calculated with the Mantel-Cox test. Hazard Ratios were calculated with the Mantel–Haenszel test (untreated/treated).

## 3. Results

### 3.1. Construction of a SV Vector Expressing Both αOX40 and IL-12

**Figure 1:**
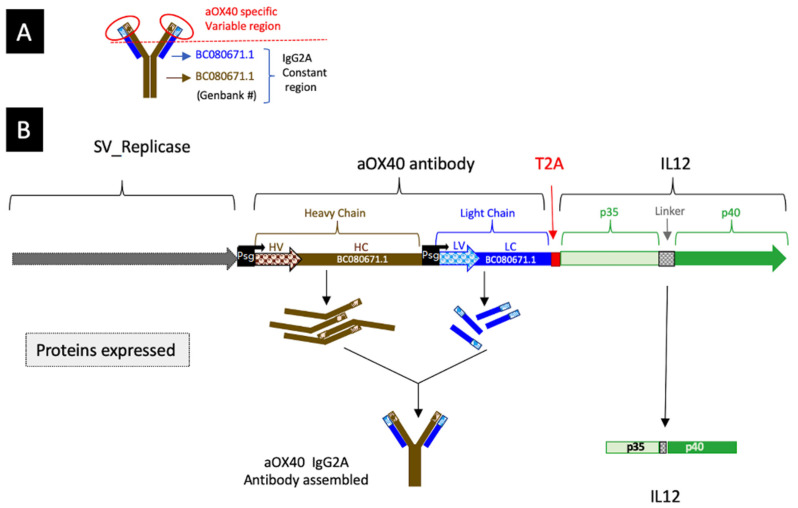
SV Replicon SV.IgGOX-40.IL-12. (**A**) Anti-OX40 IgG 2A design. (**B**) SV.IgGOX40.IL-12 linear map. The ⍺OX40 heavy chain is expressed from one SV subgenomic promoter (Psg). The ⍺OX40 light chain and IL12 cytokine are expressed from a second SV subgenomic promoter (Psg). To produce IL-12, a T2A peptide sequence from *Picornaviridae* virus family was inserted at the 3′end of ⍺OX40 light chain after removing the stop codon and before the IL-12 sequence.

A diagram and description of SV.IgGOX40.IL-12 is shown in Figure 1. An additional subgenomic promoter was inserted into the vector to express the antibody light-chain and IL-12 cytokine sequence as a multicistronic transcript. As more than two multiple subgenomic promoters have been found to yield inefficient transcription, the generation of separate antibody light-chain and IL-12 proteins was achieved by inserting a 2A peptide sequence from the *Picornaviridae* virus family at the 3′end of the αOX40 light chain after removing the stop codon and before the IL-12 sequence. The T2A motif mediates ribosomal “skipping” by preventing formation of a peptide bond between glycine and proline [71].

**Figure 2:**
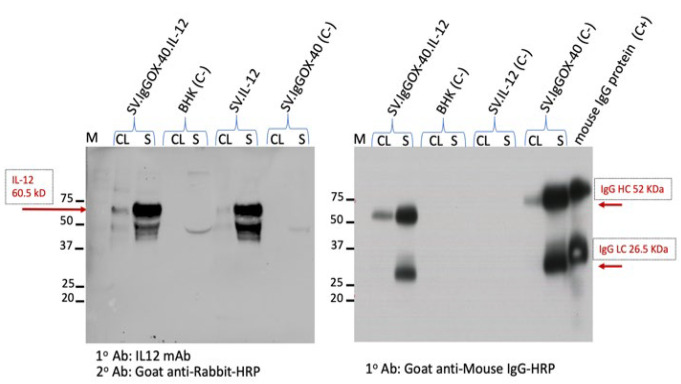
Expression of IL-12 (left) and αOX40 IgG (right) from SV.IgGOX40.IL-12. Western blots were performed for cell lysates (CL) and supernatant media (S) from BHK cells electroporated with SV.IgGOX-40.IL-12 mRNA, SV-expressing IL12, or ⍺OX40 as positive controls. Unelectroporated BHK cell line was used as negative control. M: molecular weight standard. **Left panel** shows blot incubated with mAb to IL-12 and **right panel** shows blot after incubation with Ab to IgG; commercial mouse IgG antibody served as positive control. After IgG-HRP incubation, blots were developed by chemiluminescence. Blots show expression of the correct size bands.

**Figure 3:**
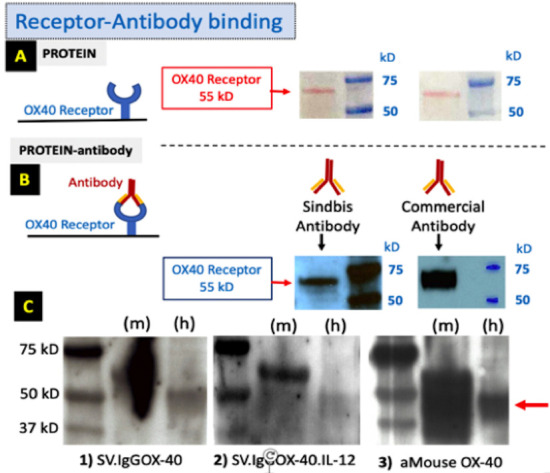
Sindbis ⍺OX40 Ab binding. (**A**) Purified OX40 receptor protein is on the Western blot membrane. (**B**) Binding to the receptor of αOX40: **left**, supernatant of Sindbis infected cells containing ⍺OX40 Ab; **right**, commercial αOX40 clone OX-86 obtained from BioXCell. (**C**) αOX40 Ab produced by Sindbis vectors (1) SV.IgGOX40; (2) SV.IgGOX40.IL-12); and (3) commercial Ab bind to mouse (m) and human (h) OX40 receptor proteins.

Proper expression of the full ⍺OX40 antibody from SV (Figure 2 right) and T2A to produce IL-12 is shown in Figure 2, left panel. Its affinity for the OX40 receptor (Figure 3A,B) was validated by Western blot. Additionally, the antibody was selected to have affinity for the mouse and human OX40 receptors (Figure 3C).

### 3.2. SV Vector Platform Cures Established Ovarian Cancer Tumors In Vivo

**Figure 4:**
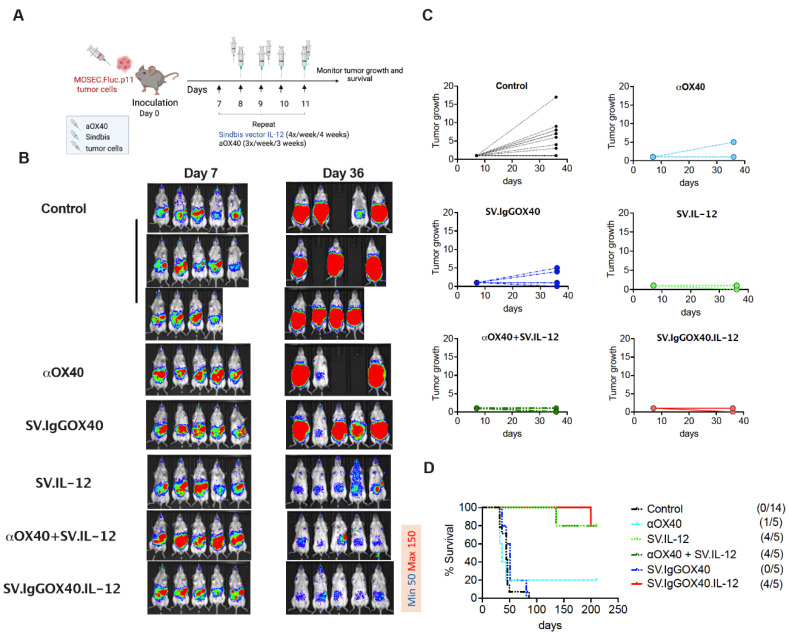
SV vector platform significantly suppresses established ovarian cancer tumors in vivo. (**A**) Experimental protocol for the ovarian cancer model used in (**B**–**D**). C57BL/6 mice were given an i.p. injection of SV.IL-12, SV.IgGOX40, SV.IgGOX40.IL-12, and/or αOX40 at the indicated times after injection of 2.5 × 10^6^ MOSEC.Fluc.p11 cells on day 0. (**B**) Representative bioluminescence images of control and treated tumor-bearing mice. (**C**) MOSEC.Fluc.p11 tumor growth curves shown as bioluminescence (RLU) (RLU day X/ RLU first day) over time for each mouse. Each line represents an individual mouse. Top left graph: control (*n* = 14), middle left graph: SV.IgGOX40 (*n* = 5), bottom left graph: αOX40+SV.IL-12 (*n* = 5). Top right graph: αOX40 (*n* = 5), middle right graph: SV.IL-12 (*n* = 5), bottom right graph: SV.IgGOX40.IL-12 (*n* = 5). (**D**) Survival plot of control and treated mice bearing peritoneally disseminated MOSEC.Fluc.p11tumors. Hazard ratios (untreated/treated) were determined with the Mantel-Haenszel method. SV.IgGOX40 (1.837), αOX40+SV.IL-12 (10.5), αOX40 (0.9921), SV.IL-12 (10.5), SV.IgGOX40.IL-12 (10.5). Results are representatives of at least two independent experiments.

As a model for high-grade serous ovarian cancer, we used C57BL/6 mouse ovarian surface epithelial cells (MOSEC/ID8) [60] that we made more stable in vivo through passaging to obtain MOSEC.p11. The MOSEC.Fluc.p11 cell line contains transfected firefly luciferase (Fluc) to monitor tumor growth using an in vivo imaging system (IVIS); we and others have observed that Fluc expression does not alter tumor engraftment and growth or the immune profile of C57BL/6 mice as shown in all untreated experiment control groups in each of our in vivo experiments [72]. Intraperitoneal injection of 2.5 × 10^6^ MOSEC.Fluc.p11 cells into syngeneic C57BL/6 albino mice gave rise to observable tumors within 7 days, at which point the treatment protocol began (Figure 4A). The efficacy of SV.IgGOX40.IL-12 was compared with SV vectors expressing IL-12 or αOX40 alone and direct injection of OX40 antibody with or without SV.IL-12 (Figure 4). IVIS signals from MOSEC.Fluc.p11 tumors are shown on day 7, before treatment, and at 36 days (Figure 4B). Photon flux quantitation indicated substantial tumor growth in untreated control mice and mice directly injected with OX40 antibody or αOX40 expressed from SV vectors, whereas SV.IgGOX40.IL-12 and SV.IL-12, with and without αOX40 antibody injection, suppressed tumor growth (Figure 4C). These results were mirrored in survival studies where 5/5 mice were alive at 150 days following treatment with SV.IgGOX40.IL-12 and 4/5 mice treated with SV.IL-12 and SV.IL-12 plus OX40 antibody (Figure 4D). OX40 antibody alone did not effectively eliminate the MOSEC-Fluc.p11 tumors.

**Figure 5:**
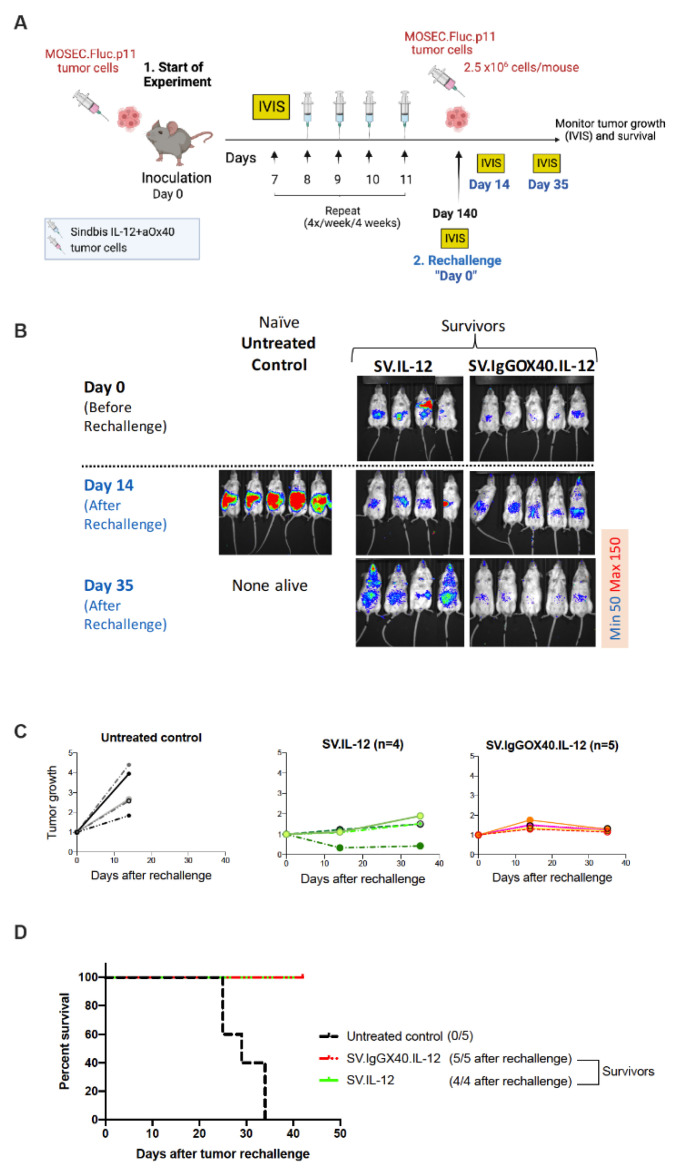
Survivors rechallenged with ovarian tumor cells are protected from tumor recurrence. (**A**) Experimental protocol for the ovarian cancer model used in (**B**–**D**). C57BL/6 mice were given an i.p. injection of SV.IL-12 or.SV.IgGOX40.IL-12 at the indicated times after injection of 2.5 × 10^6^ MOSEC.Fluc.p11 cells on day 0. Survivor treated mice were given another injection of 2.5 × 10^6^ MOSEC.Fluc.p11 cells at day 140. At this time (rechallenge day 0), naive control mice were also injected with MOSEC.Fluc.p11 cells. (**B**) Representative bioluminescence images of control and treated tumor bearing mice. (**C**) MOSEC.Fluc.p11 tumor growth curves shown as fold changes relative to luminescence on day 0 of the same mouse (RLU day X/ RLU first day). Each line represents an individual mouse. (**D**) Survival plot of control and treated mice bearing peritoneally disseminated MOSEC.Fluc.p11 tumors.

To examine the long-term effects of SV vector therapy, mice were injected with MOSEC-Fluc.p11, and on day 7 treatment was started with SV.IL-12 or SV.IgGOX40.IL-12, as shown in Figure 5A. After 140 days, survivor mice were i.p. rechallenged with MOSEC.Fluc.p11 cells (day 0) and imaged at days 14 and 35 (Figure 5B). Naive control mice, inoculated with tumor cells at the same time as the rechallenged mice, succumbed after 35 days. While SV.IgGOX40.IL-12 provided the most protection against rechallenge with no tumor growth (Figure 5C) and 100% survival (Figure 5D), SV.IL-12 was also effective. The results indicate that the SV.IgGOX40.IL-12 platform can eradicate MOSEC intraperitoneal tumors and provide long-term protection. In this model SV.IL-12 also plays an important role in treatment.

### 3.3. Therapeutic Efficacy of SV.IgGOX40.IL-12 Is Dependent on CD4 and CD8 T Cells

**Figure 6:**
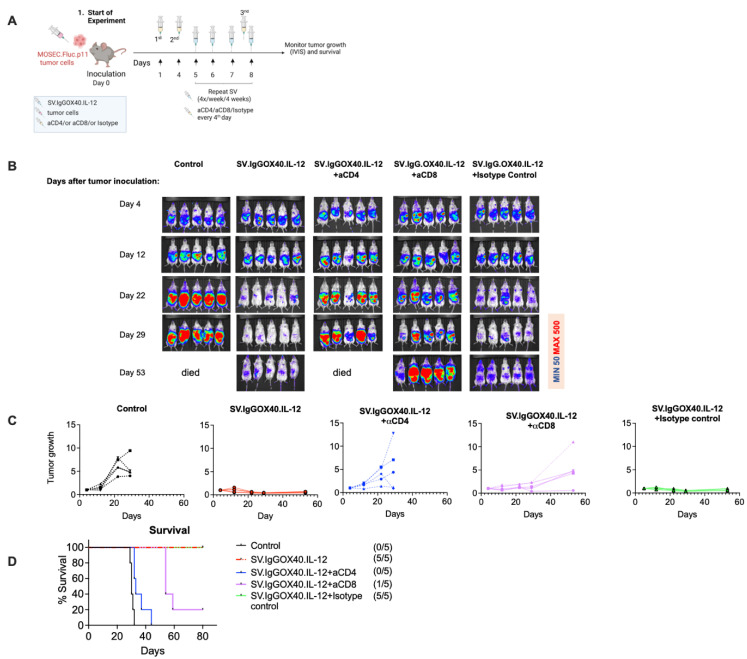
Therapeutic efficacy of SV.IgGOX40.IL-12 is dependent on CD4 and CD8 T cells. (**A**) Experimental set-up and injections. Tumors were inoculated on day 0 and SV.IgGOX40.IL-12 treatment began on day 5. Mice were injected with αCD4 (0.5 mg) or αCD8 (0.1 mg)-depleting antibody. As control, rat IgG2b (0.5 mg) isotype control was used, starting on day 1 and 4 after tumor inoculation and every 4th day for a total of 4 weeks. C57BL/6 albino mice were inoculated with 2.5 × 10^6^ MOSEC.p11.Fluc tumor cells on day 0. Mice were left untreated or were treated with SV.IgGOX40.IL-12 four days per week. (**B**) Representative bioluminescence images of control and treated tumor-bearing mice groups. (**C**) MOSEC.Fluc.p11 tumor growth curves shown as bioluminescence (RLU day X/RLU first day) total counts over time for each individual mouse. (**D**) Survival plots.

The role of T cells in MOSEC.Fluc.p11 treatment with SV.IgGOX40.IL-12 was assessed by in vivo antibody depletion of CD8 and CD4 T cells (Figure 6). Details of the treatment protocol are shown in Figure 6A. Mice were examined by IVIS on days 4, 12, 22, 29, and 53 after tumor inoculation (Figure 6B). By day 22, untreated mice show advanced tumor growth, while SV.IgGOX40.IL-12 treated mice show little or no MOSEC.Fluc.p11 tumors.Depletion of CD4 and CD8 T cells, however, diminishes SV.IgGOX40.IL-12 treatment (Figure 6B,C) indicating the importance of T cell immune response to the therapeutic process. Isotype control antibody did not inhibit the effect of SV.IgGOX40.IL-12 treatment. Increased tumor growth is greater when CD4 T cells are depleted. We have previously observed that SV.IL-12 and ⍺OX40 elevate and sustain cytotoxic CD4 T cells [7]. In mice, in which CD8 T cells are depleted, CD4 T cells can account for the slower tumor growth. 

### 3.4. Sindbis Virus Vectors Induce Increase in Tumor Infiltrating T Cells

**Figure 7:**
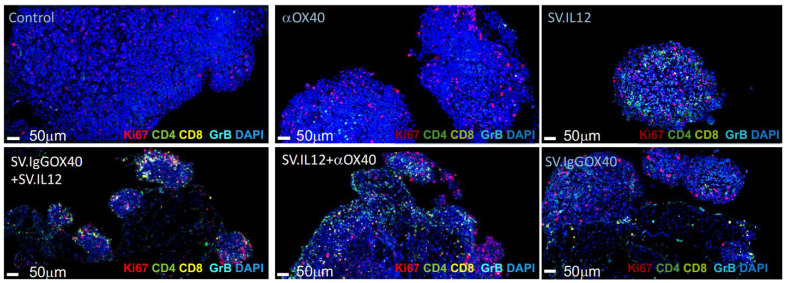
Tumors of mice treated with armed Sindbis vectors expressing IL-12 or αOX40 display increased tumor-infiltrating immune T cells. Intra-tumoral T-cell immune responses were assessed by multiplex immunofluorescence staining in tumors isolated from tumor-bearing mice. MOSEC.Fluc.p11 tumor-bearing mice were left untreated, or were treated with αOX40, SV.IL-12 (upper lane left to right) or SV.IgGOX40+SV.IL-12 (50% injection mix), SV.IL-12+αOX40, or SV.IgGOX40 single-vector (lower panel left to right). Representative images of T-cell infiltration are shown for indicated groups. Proteins of interest were stained and are indicated by color in each image: Ki-67 (red), CD4 (green), CD8 (yellow), granzyme B (turquoise), and DAPI nuclear staining appears in blue.

Our previous results indicate that IL-12 produced from SV vectors acts locally without affecting IL-12 plasma levels [7]. We, therefore, analyzed the effects of SV.IL-12, with and without αOX40 antibody, on the intratumor T-cell immune responses using multiplex immunofluorescence of fixed and sectioned MOSEC.Fluc.p11 tumors excised at 14 days after tumor implantation and 7 days after start of treatment (Figure 7). We examined the influx of CD4 and CD8 T cells, presence of the cytotoxic T cell and NK cell markers, granzyme B, and the cell proliferation marker, Ki67 (Figure 7). Untreated control tumors appeared more intact and denser and did not show the presence of T cells. Histological differences in tumor tissue are observed after OX40 antibody delivery by injection compared with SV vector delivery (SV.IgGOX40). Tumor tissue from mice that received injected αOX40 was denser, showed proliferation markers, and some granzyme B, suggesting the presence of cytotoxicity or NK cells although T cells were not overtly observed. Tumors from SV.IgGOX40-treated mice showed significant cell lysis and the presence of CD8 T cells and granzyme B. It is likely that greater amounts of αOX40 could be delivered to the tumor by SV infection that can also produce a proinflammatory response. SV.IL-12 plus injected OX40 antibody and SV.IL-12 alone shows similar patterns of cell lysis, T cell influx, and granzyme B. However, administration of equal amounts of SV-vector-expressing IL-12 and αOX40 results in greater cell lysis and influx of immune cell markers. The local delivery of both immunomodulators by the oncolytic SV vectors yield an enhanced response that has been observed in other cancer models [7].

### 3.5. Treatment-Induced Metabolic Reprograming of T Cells

**Figure 8:**
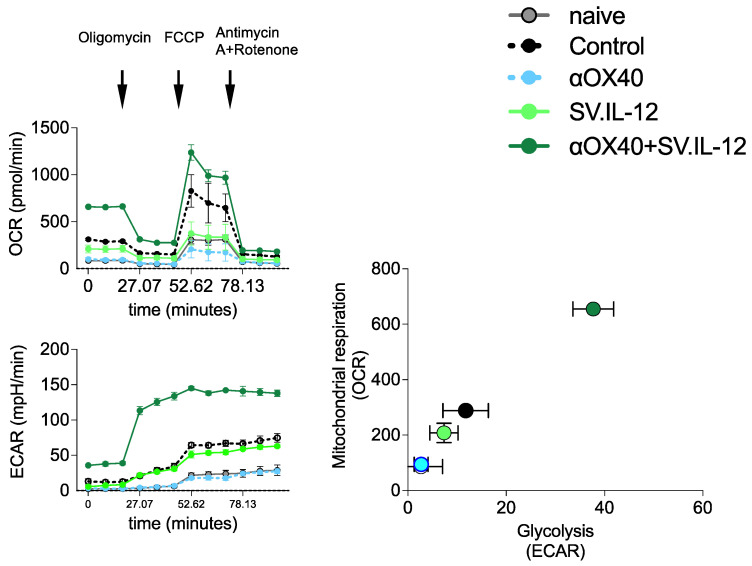
Metabolic impact on T cells from mice treated with combination therapies that include SV.IL-12 plus αOX40 antibody in the MOSEC model. Tumor-bearing mice were left untreated or treated with SV.IL-12 and/or αOX40. T cells were isolated from spleens on day 7 after treatment starts (day 14 from tumor implantation). (**A**) Mitochondrial respiration was assessed by measuring the median values of (**A**) oxygen consumption rates (OCRs) and (**B**) baseline extracellular acidification rates (ECARs) in T cells of indicated groups using an extracellular flux analyzer on day 7 of treatments. Oligomycin, FCCP, antimycin A, and rotenone were injected as indicated to identify energetic mitochondrial phenotypes. (**C**) Energy profile (OCRs versus ECARs) of T cells from naive or MOSEC.Fluc.p11 tumor-bearing mice treated with SV.IL-12 and/or αOX40. Error bars indicate SEM. Note that the αOX40 and naive graph points are super-imposed. Results are representatives of at least two independent experiments.

T cells must adapt metabolically to overcome a repressive tumor microenvironment [73,74,75,76]. Our previous studies showed that the combination of IL-12 and αOX40 was able to provide metabolic support for T cell anti-tumor activity in two other cancer models [7]. We examined the metabolic profile of splenic T cells from naive mice, the MOSEC.Fluc.p11 tumor-bearing untreated mice, and mice treated with αOX40 alone and SV.IL-12 with and without αOX40 antibody (Figure 8). After 14 days of tumor implantation and 7 days of treatment, mitochondrial oxidative consumption rates (OCR) and extracellular acidification rates (ECAR) of T cells were measured using Agilent Seahorse analysis. T cells from mice with combined treatment of IL-12 and αOX40 had a higher OCR rate compared with all other groups (Figure 8A). Residual ATP production after addition of oligomycin, which inhibits mitochondrial ATP synthetase, suggests that glycolytic activity occurs in T cells from combined treatment, which is also shown by ECAR measurements (Figure 8B). Inhibition of electron transport with FCCP (Figure 8A) stimulates maximum respiration by signaling an energy demand. T cells from combined treatment show the highest maximal respiration rate. Addition of antimycin A and rotenone reduces respiration to a minimum rate of non-mitochondrial oxygen consumption. The OCR profile shows that T cells from mice treated with both IL-12 and OX40 antibody, compared with other treatment groups, contain spare respiratory capacity (maximal respiration − basal) that can correlate with an enhanced anti-tumor response. The increase in OCR in T cells from untreated control mice is not clearly understood but may result from greater stimulation of T cells due to higher tumor burden at this 7-day treatment time point. The OCR/ECAR profile (Figure 8C) distinctly shows a more basal energetic state for T cells from mice treated with both OX40 antibody and SV.IL-12.

### 3.6. SV Vector Therapy Selectively Remodels Tumor Transcriptomes in Tumor-Bearing Mice

**Figure 9:**
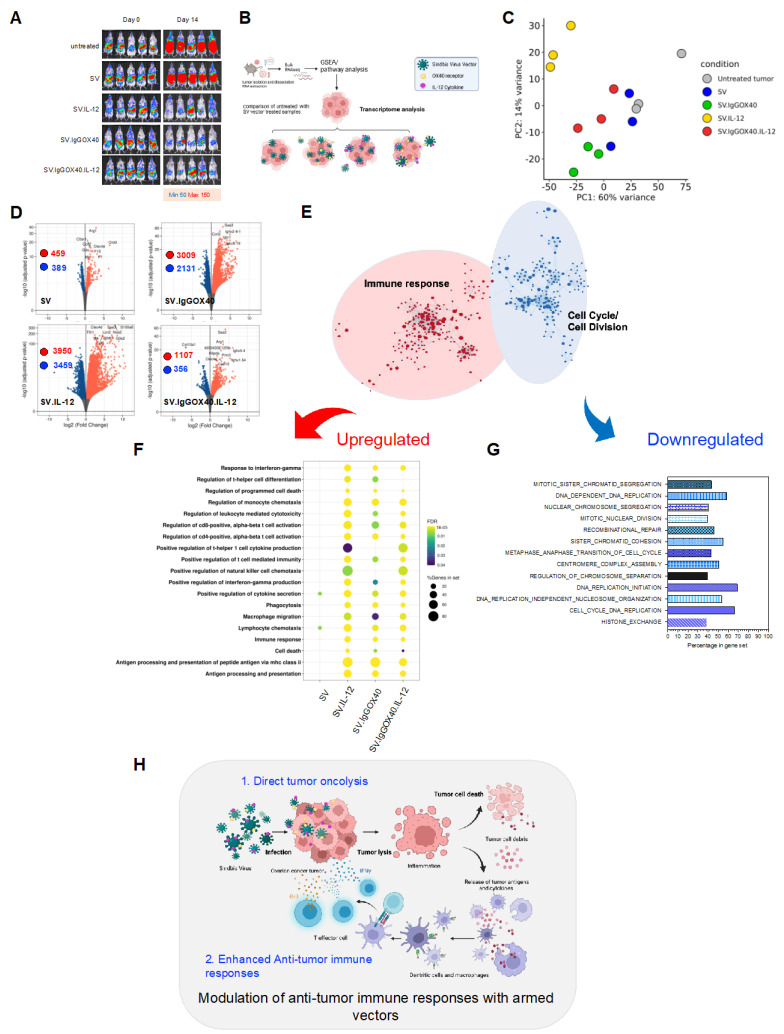
Sindbis virus vector therapy remodels tumor transcriptomes in tumor-bearing mice. Potent and targeted anti-tumor SV therapy in vivo for ovarian cancer. Sindbis virus vector therapy remodels tumor transcriptome in tumor-bearing mice. We performed RNAseq to evaluate SV-therapy-dependent inhibition of tumor growth in tumor-bearing mice within the first 2 weeks of treatment. We characterized functional changes in gene expression with regards to treatments using GSEA on transcriptomes of tumors isolated from untreated and SV-treated mice. (**A**) Inhibition of tumor growth visualized by IVIS in untreated and SV-expressing IL-12 and/or αOX40 versus empty SV-vector-treated tumor-bearing mice during the first 2 weeks of treatments. (**B**) Experimental set-up. (**C**) PCA on transcriptome of tumor tissue isolated from the indicated treatment groups and controls (*n* = 3 mice/group). (**D**) Volcano plots indicating DEGs (*p*-adjusted < 0.05) in tumors isolated from mice treated with SV Empty (SV), SV.IL-12, SVIgGOX40, or SV.IgGOX40.IL-12 compared to untreated (control). (**E**) Pathway and network analysis. Upregulated (**F**, red) and downregulated (**G**, blue) molecular pathways are shown. (**F**) Dotplot visualization of upregulated pathways related to immune response in tumors of unarmed and armed SV-treated mice compared to untreated. Pathway enrichment analyses of DEG (log_2_FC > 1; *p* < 0.05) were performed using STRING against the GO dataset for biological processes. The color of the dots represents the false discovery rate (FDR) value for each enriched GO term, and size represents the percentage of genes enriched in the total gene set. (**G**) Selected GO terms of top 20 enriched downregulated biological pathways based on GSEA (NES < −2.45, negatively correlated; FDRq = 0; *p*NOM = 0; *p*FWER = 0) of DEGs in unarmed SV-treated compared to untreated tumors. (**H**) Modulation of anti-tumor immune responses by armed Sindbis viral vectors.

One of the most prominent and first identified features of ovarian cancer tumors in SV-vector-treated tumor-bearing mice is tumor growth inhibition (Figure 9A). To evaluate the underlying treatment dependent alterations of ovarian tumor physiology we performed RNA sequencing (RNA-seq) followed by STRING [69] and gene set enrichment analysis (GSEA) [68]. We compared differentially expressed genes (DEGs) on tumor tissue isolated from mouse groups treated with empty SV and gene-expressing SV vectors (IL-12, OX40 antibody, or SV.IgGOX40.IL-12) and controls after 1 week of treatment (Figure 9B). Principal component analysis (PCA) [67] highlighted distinct gene expression profiles of tumors across all treatment groups and untreated controls. (Figure 9C). We compared DEGs (*p*-adjusted < 0.05) of untreated tumors versus tumors treated with empty or gene-expressing SV vectors; 485 DEGs were upregulated and 389 were downregulated in the SV empty vector group. In contrast, 3009 DEGs were upregulated and 2131 were downregulated in samples treated with SV-expressing αOX40, and 3950 DEGs were upregulated and 3459 were downregulated in samples treated with SV-expressing IL-12. Interestingly, a slightly lower number of 1107 upregulated and 356 downregulated DEGs in samples treated with SV-co-expressing αOX40 and IL-12 compared with single-agent SVs was observed (Figure 9D). Overall, the lower numbers of down- and upregulated DEGs in tumor samples treated with empty SV vector versus untreated controls compared with all other groups indicates successful delivery and expression of αIgGOX40 and IL-12 by SV vectors at the tumor site.

Pathway and network analysis (Figure 9E–G) of the RNAseq data against the Gene Ontology (GO) dataset for biological processes suggest that tumor cells are eliminated because their transcriptional signature becomes associated with marked downregulation of gene transcripts that are important in DNA replication, RNA transcription, and cell division. Gene set enrichment analysis of DEGs from untreated control versus SV vectors treatments was performed to characterize the functional changes in gene sets related to downregulated pathways in the different groups (Figure 9G) indicating direct tumor killing mechanisms by SV vectors. Upregulated pathways are primarily those involved in mounting a potent immune response (Figure 9F) and are likely expressed from immune cells that have infiltrated the tumors, as indicated by pathway analysis of DEGs (log_2_FC > 1; *p* < 0.05) via STRING, which we used to investigate the modulation of anti-tumor immune responses to SV vector therapies. Gene sets related to biological pathways regulating innate and adaptive anti-tumor immune responses, for example, regulation of monocyte chemotaxis, macrophage migration, regulation of natural killer cell chemotaxis, antigen processing and presentation, positive regulation of T helper cytokine production regulation of CD4- and CD8-positive alpha, beta T-cell activation, and positive regulation of IFN*γ* production, were observed in the treated tumor samples when compared with untreated controls. Empty SV vector treatment elicits very little gene expression related to immune function compared with genes expressed in SV-vector-treated groups. Arming SV vectors with selective immunostimulatory molecules underlines the potential of SV vector platform as an adaptive and promising therapy for improving treatments for different types of cancers.

## 4. Discussion

We have developed an SV vector platform that combines the expression of the cytokine, IL-12, and an agonistic antibody to the co-stimulatory receptor OX40. Our previous studies have shown that SV.IL-12 combined with systemically delivered OX40 antibody provides successful therapy for colon and prostate cancer mouse models [7]. SV vectors represent an alternative to many oncolytic vectors. Most oncolytic vectors used today are administered intratumorally [77], owing to barriers which prevent systemic targeting, including dilution of the virus in the bloodstream, neutralization by anti-viral antibodies and complement proteins, virus particle sequestration in liver Kupffer cells and splenic macrophages, and the limited permeability of tumor neovessels [78,79]. These barriers do not impact systemic administration of SV to a significant degree [9,80]. As a blood-borne virus with a relatively long half-life [8,81], SV vectors can be administered systemically and reach tumor cells throughout the body, targeting tumors without infecting normal tissues [3,4,19,61,80]. SVs can target many xenografts, syngeneic, and spontaneous tumors [3,4,61] and show antitumor activities in multiple preclinical models [3,4,19,61,80]. Further, translation to clinical application is feasible as we can purify SV vectors and produce them under GMP conditions to very high titers (>10^11^ TU/mL), which helps compensate for dilution in the bloodstream. SV (average diameter 60–70 nm) is smaller than most viruses (e.g., adenovirus 90–100 nm, vesicular stomatitis virus 65–185 nm, and lentivirus 95–175 nm). Combined with its natural blood-borne capability, which many other vectors lack, the smaller size makes SV vectors suitable for systemic delivery.

A single SV vector has numerous advantages, including dosing, cost, and manufacturing, over the combination of SV.IL-12 and αOX40 or even two separate vectors one encoding IL-12 and the other encoding αOX40. In this study we used a MOSEC.Fluc.p11 cell line as a model for high-grade serous ovarian carcinoma, a lethal ovarian cancer. Unlike colon and prostate cancer treatment, in which combined SV.IL-12 and OX40 antibody clearly was most efficacious, MOSEC.Fluc.p11 tumors were eradicated almost as well with either SV.IL-12 or SV.IgGOX40.IL-12 vectors. However, mice treated with SV.IgGOX40.IL-12 vectors appear to be better protected from tumor rechallenge suggesting that αOX40 with IL-12 may be important for generation of long-term T-cell memory.

In agreement with previous results, the therapeutic effect of SV vectors armed with IL-12 and/or αOX40 was found to be dependent upon both CD8 and CD4 T cells. T-cell infiltration into MOSEC.Fluc.p11 tumors was shown by immunohistochemistry. Immunofluorescence of T cells and T-cell activation markers were clearly observed when SV vectors were used for treatment. T-cell activity and cell lysis were mostly observed in the presence of SV vector IL-12 and αOX40. The ability of SV vectors to infect the tumors, induce apoptosis, release tumor antigens and stimulate an immune response is evident. In Figure 9H we illustrate these processes and show the major transcriptional changes that occur in the tumors (Figure 9).

Combination of SV vectors expressing IL-12 and αOX40 distinctly confer metabolic enhancement to T cells. The OCR/ECAR profile of T cells from mice treated with both αOX40 and SV.IL-12 showed both a higher basal energetic state and spare respiratory capacity. This altered metabolic state of T cells by SV.IL-12 and the OX40 antibody was also observed in our previous studies [7]. Enhanced metabolic activity and migration of T cells act to overcome a repressive tumor microenvironment. OX40 signaling increases the survival capacity of effector T cells and decreases suppressive T-regulatory cells [33]. IL-12 increases the expression of OX40 on CD4 T cells and stimulates the production of IFNγ [46,47]. In a related study, we have also shown that OX40 antibody administered with SV.Spike elicits long-lasting neutralizing antibodies and a vigorous T-cell response in mice that protects against authentic SARS-CoV-2 virus [82]. The expression of OX40 receptor on CD4 T cells is interesting with respect to the T-cell depletion studies (Figure 6). Removal of CD4 T cells has a more adverse effect on SV.IgGOX40.IL-12 therapy, suggesting that cytotoxic CD4 cells may play an important role.

We present the SV.IgGOX40.IL-12 vector as a cancer immunotherapy platform. Previous studies and the results shown here, for MOSEC.Fluc.p11 tumors, indicate that the combined SV and immunomodulatory molecules, IL-12 and αOX40, provide curative, long-term therapy in mouse models. Differences have been observed, however, in the treatment process. Mouse CT26 colon cancer tumors are not infected by SV yet are successfully treated with SV.IL-12 and OX40 antibody administered one time per week for 4 weeks, whereas MyC-CaP prostate tumors, which are susceptible to SV infection, require four injections per week for 4 weeks to eliminate tumor growth [7]. In this study, SV.IL-12 was almost equivalent to SV.IgGOX40.IL-12 for treatment of MOSEC.Fluc.p11 tumors. Several factors may account for the variations in treatment effects. Each type of tumor most certainly has distinctive microenvironments (TME) presenting different challenges to treatment. MOSEC tumors are also affected by ascites fluid having its own pathophysiology [1]. The cancer mouse models in our investigations are of different mouse strains having backgrounds that can affect their immune response, including the level of pre-existing T-cell immune repertoires. In this MOSEC-Fluc.p11 tumor model, we examined T cells from tumor tissue as opposed to peripheral T cells. In future studies we will focus on comparison of peripheral and intratumoral immune cells and single-cell RNAseq, which will be more informative. Combining these data with cytokine kinetics in tumor and ascites of treated mice will improve our understanding of the mechanism of SV.IgGOX40.IL-12. therapy. 

In conclusion, while oncolytic viruses expressing heterologous genes have been generated by others, none, to our knowledge, encodes both IL-12 and αIgGOX40. Nor can they markedly change the transcriptome signature and metabolic program of T cells, leading to the development of highly activated, terminally differentiated, effector T cells with enhanced capacity to infiltrate the repressive TME and kill tumors or generate protective memory T cells. Additionally, Sindbis SV vectors can systemically target tumors and their metastases throughout the body, unlike most agents, such as antibodies, that need to be directly administered into the tumor sites. Finally, SV.IgGOX40.IL-12 does not require prior knowledge of tumor antigen specificity. We promote SV.IgGOX40.IL-12 as a safe and effective therapy for multiple types of cancer.

## Data Availability

All sequencing data that support the findings of this study will be deposited in the National Center for Biotechnology Information Gene Expression Omnibus (GEO) and are accessible through the GEO Series accession number that will be provided and including all other relevant data included in the article, and further inquiries can be directed to the corresponding authors. Accession numbers have not yet been obtained at the time of submission, but they will be provided during review, prior to publication.
